# First report of naturally infected *Sergentomyia minuta* with *Leishmania major* in Tunisia

**DOI:** 10.1186/s13071-015-1269-4

**Published:** 2015-12-21

**Authors:** Kaouther Jaouadi, Wissem Ghawar, Sadok Salem, Mohamed Gharbi, Jihene Bettaieb, Rihab Yazidi, Mariem Harrabi, Omar Hamarsheh, Afif Ben Salah

**Affiliations:** Department of Medical Epidemiology, Laboratory of Transmission, Control and Immunobiology of Infections (LR11IPT02), Institut Pasteur de Tunis, 13 Place Pasteur BP-74, Tunis-Belvedere, 1002 Tunisia; Laboratory of Parasitology, National School of Veterinary Medicine, University of Manouba, 2020 Sidi Thabet, Tunisia; Department of Biological Sciences, Faculty of Science & Technology, Al-Quds University, Jerusalem, Palestine

**Keywords:** *Leishmania major*, *Sergentomyia minuta*, *Phlebotomus papatasi*, Cutaneous leishmaniasis, Tunisia

## Abstract

**Background:**

Many sand fly species are implicated in the transmission cycle of *Leishmania* parasites around the world. Incriminating new sand flies species, as vectors of *Leishmania* is crucial to understanding the parasite–vector transmission cycle in different areas in Tunisia and surrounding countries.

**Findings:**

Seventy-four unfed females belonging to the genera *Sergentomyia* and *Phlebotomus* were collected in South Tunisia between June and November 2014, using sticky papers. PCR-RFLP (Restriction Fragment Length Polymorphism) analysis of the internal transcribed spacer 1 (ITS1) was used for *Leishmania* parasites detection and identification. *Leishmania* (*L.*) *major* (Yakimoff & Shokkor, 1914) was identified within two *Sergentomyia* (*S.*) *minuta* (Rondani, 1843) and one *Phlebotomus papatasi* (Scopoli, 1786).

**Conclusion:**

This is the first report of *L. major* identified from *S. minuta* in Tunisia. This novel finding enhances the understanding of the transmission cycle of *L. major* parasites of cutaneous leishmaniasis in an endemic area in South Tunisia.

## Findings

In Tunisia and other Old World countries, sand fly species of the genus *Phlebotomus* are traditionally regarded as the proven vectors of *Leishmania* parasites. However, a few studies have recently suggested the possible involvement of some species of the genus *Sergentomyia* in the transmission of *Leishmania,* particularly in some African countries [1–3]. There *Sergentomyia* spp. are the predominant sand fly taxa, and appear able to tolerate different biotopes and environmental conditions. Although, *Sergentomyia* spp. are proven vectors of reptile *Leishmania* spp. [4] which are non-pathogenic to humans, and at least some of them feed on humans and/or mammalian reservoirs which can contain *Leishmania* spp. pathogenic to humans [5]. Therefore, their role as vectors in some visceral and cutaneous leishmaniasis foci where *Sergentomyia* spp. were abundant is suspected. Thus, the exact role of *Sergentomyia* spp. in transmitting mammalian leishmaniasis remains to be clarified [1, 2, 5–7].

An epidemiological study was carried out in Gafsa governorate in southwestern Tunisia, an old emerging focus of cutaneous leishmaniasis (CL), with the objective of detecting and characterizing novel vectors of *Leishmania* parasites in the region.

Sand fly collections were collected from June to November 2014, using sticky papers. Verbal informed consent was obtained from residents in each of the community. Specimens were sorted and transferred to 70 % ethanol before being processed. Species identification was determined using the available morphological keys for sand fly in Tunisia [8]. Sand flies abdomens were transferred to individual sterilized 1.5 ml vials and stored at −20 °C. DNA from each individual female was extracted using the QIAamp® DNA Mini Kit (QIAgen, Germany) according to the manufacturer’s instructions. The presence of the *Leishmania* DNA was tested by the amplification of the ribosomal internal transcribed spacer 1 (ITS1) of these parasites and followed by Restriction Fragment Length Polymorphism (RFLP) analysis in which the ITS1-PCR products were digested by *Hae*III restriction enzyme using protocol previously described [9]. The following WHO reference strains of *Leishmania* (*L.*) *major*, *Leishmania killicki* (Rioux, Lanotte & Pratlong, 1986) and *Leishmania infantum* (Nicolle, 1908) were used as positives controls: *L. major* MON-25 MHOM/TN2009/S600, *L. killicki* MON-8 MHOM/TN/2011/MX and *L. infantum* MON-1 MHOM/TN/94/LV50.

In total, 325 sand flies were captured and identified (190 males and 135 females). According to morphological identification, three species belonging to each genus *Phlebotomus* and *Sergentomyia* were identified: 162 *Phlebotomus* (*P.*) *papatasi* (Scopoli, 1786), 16 *Phlebotomus* (*P.*) *(Larroussius) longicuspis* (Nitzulescu, 1930), 5 *Phlebotomus* (*P.*) (*Larroussius*) *perniciosus* (Newstead, 1911), 67 *Sergentomyia* (*S.*) *minuta* (Rondani, 1843), 71 *Sergentomyia* (*S.*) *fallax* (Parrot, 1921) and 4 *Sergentomyia* (*S.*) *dreyfussi* (Parrot, 1933).

In this study, males were not considered regarding that only females were haematophageous. Indeed, fed females were excluded from the analysis because the presence of *Leishmania* DNA in these specimens can be an immediate consequence of blood feeding on an infected host. Only seventy-four unfed females belonging to *S. minuta* (*n* = 24), *S. fallax* (*n* = 26), *S. dreyfussi* (*n* = 4) and *P. papatasi* (*n* = 20) were tested for the presence of *Leishmania* parasites DNA which should be considered as the result of the multiplication of the parasites in the gut of the sand fly.

**Fig. 1:**
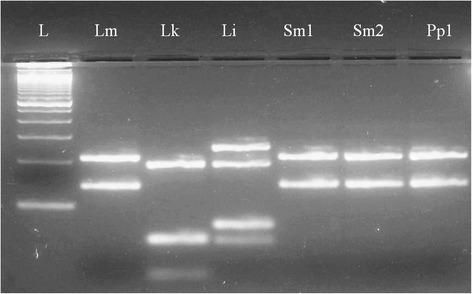
RFLP products of the amplified ITS1 fragment using *Hae*III enzyme. L: 100 bp size marker (Invitrogen®), Lm: *L. major* MHOM/TN2009/S600 (two fragments of 132 bp and 206-bp), Lk: *L. killicki* MON-8 /MHOM/TN/2011/MX (three fragments of 188-bp, 57-bp and 26-bp respectively), Li: *L. infantum* LV50 (three fragments of 187-bp, 72-bp and 55-bp respectively). Positive samples: Sm1 and Sm2 (two *S. minuta* individuals), and Pp1 (one *P. papatasi*)

Two *S. minuta* and one *P. papatasi* were found positive for *L. major* DNA using the ITS1-PCR-RFLP method (Fig. 1). The detection of *L. major* in *P. papatasi* was not unexpected as this species has already been implicated as a vector of *L. major* in Tunisia [10]. However, our study is the first report of *L. major* infecting *S. minuta* in Tunisia, North Africa.

This preliminary finding raises the speculation and highlights the involvement of *S. minuta* in the zoonotic CL transmission cycle. Our results corroborate the finding in previous reports of *L. major* detection in *S. minuta* in Portugal [7] and in *Sergentomyia ingrami* (Newstead, 1914) in Ghana [3], respectively. *L. major* and *Leishmania donovani* (Laveran & Mesnil, 1903) DNA have previously been reported in Indian *Sergentomyia* spp. [5]. Some authors have reported *Sergentomyia* spp. as a common human-biting species, including *Sergentomyia* (*S.*) *schwetzi* (Adler, Theodor & Parrot, 1929) in Sudan [11], as well as *Sergentomyia* (*Sintonius*) *clydei* (Sinton, 1928) and *Sergentomyia* (*Parrotomyia*) *africana* (Newstead, 1912) [12]. Furthermore, *Parvidens heischi* (Kirk & Lewis, 1950) (formerly considered as belonging to the *Sergentomyia* genus) is a very aggressive human biter [13]. Based on faunistic evidence, another study conducted in Senegal suggesting the implication of *S. schwetzi* and *Sergentomyia dubia* (Parrot, Mornet & Cadenat, 1945) in the transmission of canine leishmaniasis [14]. In Mali, several specimens of *Sergentomyia* (*Spelaeomyia*) *darlingi* (Lewis & Kirk, 1954) were found positive for *L. major* based on DNA screening [1]. Our results, in addition to those previously described, suggest that *Sergentomyia* species are involved in the transmission cycle of *Leishmania* parasites. Therefore, the prevailing opinion that leishmaniasis are strictly transmitted by sand flies belonging to the *Phlebotomus* genus in the Old World is naïve.

The present study demonstrated, for the first time, the detection of *L. major* DNA in *S. minuta* from an endemic area of cutaneous leishmaniasis in South Tunisia. This illustrates the potential role of *S. minuta* in the transmission cycle of *L. major* in Tunisia, adding useful information to address the key epidemiological questions on this disease control. However, further research should be directed to investigate the actual role of *Sergentomyia* species in the transmission of *Leishmania* parasites in different endemic regions including blood meal analysis and parasite isolation, on larger samples of this genus, are now needed to confirm their respective roles as a potential vectors for zoonotic CL caused by *L. major* in Tunisia.

## Abbreviations

CL: cutaneous leishmaniasis
ITS1: internal transcribed spacer 1
PCR: polymerase chain reaction
RFLP: Restriction Fragment Length Polymorphism
*S.*: *Sergentomyia*
spp.: species
